# The genetic determinants of language network dysconnectivity in drug-naïve early stage schizophrenia

**DOI:** 10.1038/s41537-021-00141-8

**Published:** 2021-03-03

**Authors:** Jingnan Du, Lena Palaniyappan, Zhaowen Liu, Wei Cheng, Weikang Gong, Mengmeng Zhu, Jijun Wang, Jie Zhang, Jianfeng Feng

**Affiliations:** 1 Institute of Science and Technology for Brain-Inspired Intelligence, Fudan University, Shanghai, China; 2 Shanghai Key Laboratory of Psychotic Disorders, Shanghai Mental Health Center, Shanghai Jiao Tong University School of Medicine, Shanghai, China; 3 Key Laboratory of Computational Neuroscience and Brain Inspired Intelligence (Fudan University), Ministry of Education, Shanghai, China; 4 Department of Psychiatry and Robarts Research Institute, University of Western Ontario, London, ON Canada; 5 Lawson Health Research Institute, London, ON Canada; 6 Psychiatric and Neurodevelopmental Genetics Unit, Center for Genomic Medicine, Massachusetts General Hospital, Boston, MA USA; 7 Department of Psychiatry, Massachusetts General Hospital, Harvard Medical School, Boston, MA USA; 8 Centre for Functional MRI of the Brain (FMRIB), Nuffield Department of Clinical Neurosciences, Wellcome Centre for Integrative Neuroimaging, University of Oxford, Oxford, UK; 9 State Key Laboratory of Cognitive Neuroscience and Learning and IDG/McGovern Institute for Brain Research, Beijing Normal University, Beijing, China; 10 Department of Computer Science, University of Warwick, Coventry, UK

**Keywords:** Biomarkers, Schizophrenia

## Abstract

Schizophrenia is a neurocognitive illness of synaptic and brain network-level dysconnectivity that often reaches a persistent chronic stage in many patients. Subtle language deficits are a core feature even in the early stages of schizophrenia. However, the primacy of language network dysconnectivity and language-related genetic variants in the observed phenotype in early stages of illness remains unclear. This study used two independent schizophrenia dataset consisting of 138 and 53 drug-naïve first-episode schizophrenia (FES) patients, and 112 and 56 healthy controls, respectively. A brain-wide voxel-level functional connectivity analysis was conducted to investigate functional dysconnectivity and its relationship with illness duration. We also explored the association between critical language-related genetic (such as FOXP2) mutations and the altered functional connectivity in patients. We found elevated functional connectivity involving Broca’s area, thalamus and temporal cortex that were replicated in two FES datasets. In particular, Broca’s area - anterior cingulate cortex dysconnectivity was more pronounced for patients with shorter illness duration, while thalamic dysconnectivity was predominant in those with longer illness duration. Polygenic risk scores obtained from FOXP2-related genes were strongly associated with functional dysconnectivity identified in patients with shorter illness duration. Our results highlight the criticality of language network dysconnectivity, involving the Broca’s area in early stages of schizophrenia, and the role of language-related genes in this aberration, providing both imaging and genetic evidence for the association between schizophrenia and the determinants of language.

## Introduction

Schizophrenia is one of the most costly and disabling neuropsychiatric disorders. Despite the large number of neuroimaging studies investigating this illness, the origins and the mechanism of schizophrenia has remained largely elusive. Mechanistic delineation is impeded by confounding effects of treatment, as well as the dynamic progression of the disease through several stages in some subgroups of affected patients. While progressive nature of brain changes has been demonstrated in this illness^1–3^, especially in patients with poor prognosis^4,5^, a number of patients recover after the first psychotic episode, and cognitive functioning does not appear to deteriorate over time^6^. Importantly, the early symptomatic years form the most crucial stage of the illness, as long term trajectory is mostly determined during this stage^3,7^. It has been suggested that prognostically critical abnormalities in functional connectivity are evident during the first two years of illness^8–10^, highlighting the critical importance of studying patients with drug-naïve first-episode schizophrenia (FES). Furthermore, the brain regions critical for the variable prognostic trajectories are likely to be captured by studying the early stages of the illness^2,11,12^.

While familial history is the largest known risk factor for schizophrenia^13^, the brain networks through which the genetic risk operates in schizophrenia remain unknown. Given that functional dysconnectivity patterns are modified with illness progression and treatment^9,14^, it is likely that the early, rather than later stage dysconnectivity, relates more to genetic factors. Patients with schizophrenia have marked language dysfunction and it has been suggested that schizophrenia has a close aetiological relationship with language^15,16^. However, it is yet unknown if language-related genetic markers influence the early stage dysconnectivity in drug-naïve first episode schizophrenia (FES) patients.

In this study we recruited two independent datasets consisting of altogether 191 drug-naïve FES patients and 168 healthy subjects. These unique datasets involved patients with illness duration ranging from 1 to 100 weeks, allowing for a dynamic analysis of disease progression. We adopted voxel-level whole-brain functional connectivity analysis to investigate the progressive functional connectivity changes in these patients that are divided into short-duration and long-duration groups based on median of illness duration. We furthermore tested whether the variations in schizophrenia-related network-level dysconnectivity is related to the genetic mutations in language-readiness genes. Given that compensatory (i.e., secondary) brain changes occur when the illness persists over time^17^, we expected genetic determinants to play a crucial role in the dysconnectivity patterns in early, but not the later stages of the illness. To this end we obtained pathway-specific polygenic risk scores (PRS) from language-related genes. Selecting a subset of genes that are grouped into the same pathway, or co-expressed with given candidate genes for calculating PRS, termed ‘pathway-specific PRS’ or biologically informed PRS^18–20^, is widely used in image genetics of psychiatric disorders including schizophrenia^21,22^. The goal of this work is to bridge the mechanistic gap between genetic variations and neuroimaging-based measure of brain-wide dysconnectivity in schizophrenia. To this end, we investigate if the dysconnectivity in language-related regions, (i.e., Broca’s area) and corresponding language-related genetic variations are related to the early stages of the disease.

## Results

### Cross-validated thalamic and IFG dysconnectivity

**Fig. 1:**
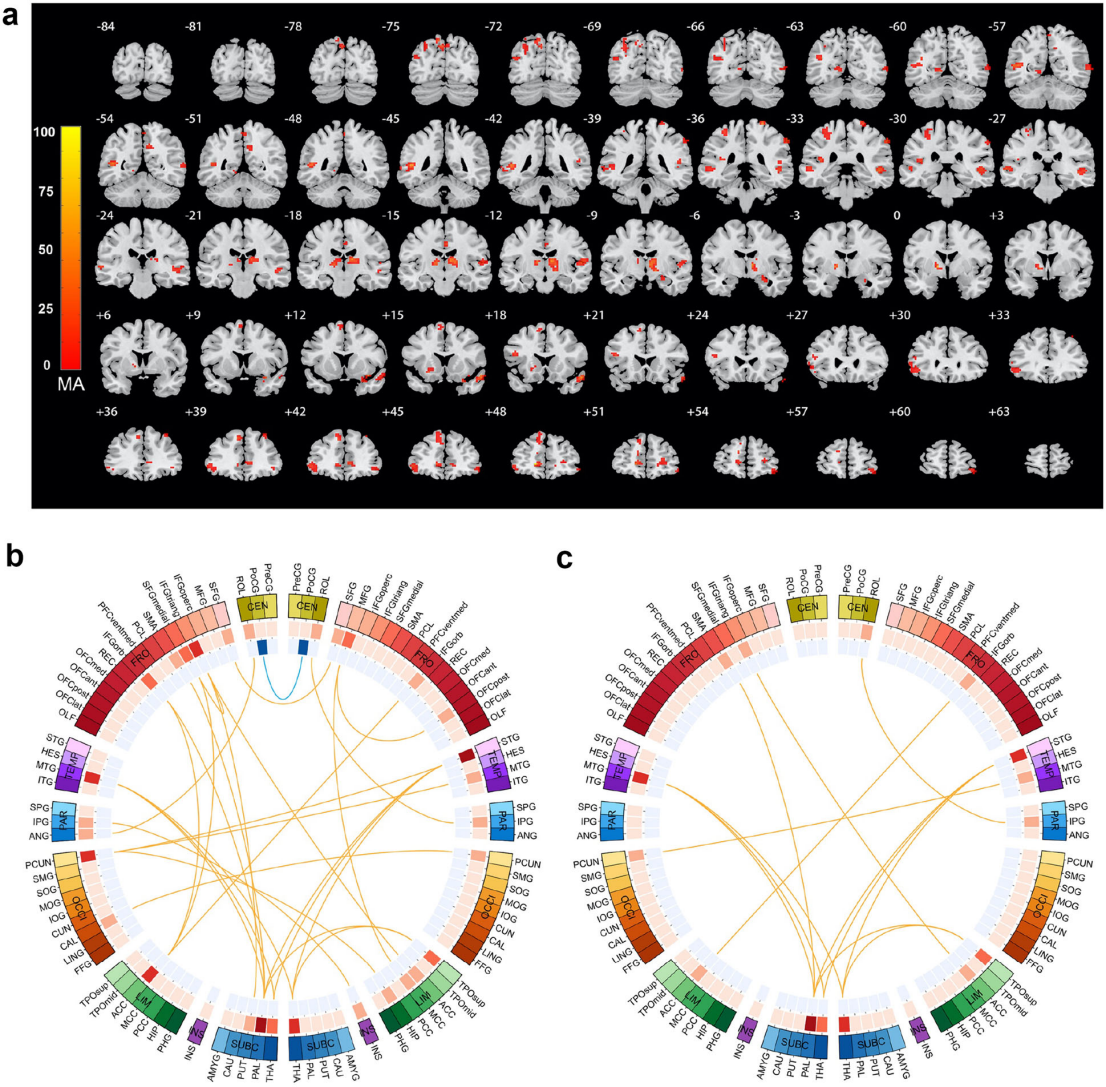
Significantly altered functional connectivity clusters (FC clusters) identified in the first-episode schizophrenia (FES) patients compared to healthy controls in the primary and validation dataset. **a** Voxels showing the largest number of voxel-level functional connectivity differences in FES patients in the primary dataset. The color bar represents the measure of association (MA) which is the number of significantly altered functional connectivities relating to each voxel. **b** Significantly altered FC clusters identified in FES patients in the primary dataset (33 FC clusters in total, details see Supplementary Table 2). The color of the 3 circles (from inside to outside) denotes: the number of deceased links; the number of increased links; the 94 different automatic anatomical labeling (AAL2) regions. Red lines mean that patients have a higher mean functional connectivity than controls, and blue lines indicate the opposite. The anatomical abbreviations in the figure are shown in the Supplementary Table 1. **c** 16 of the 33 significantly altered FC clusters were replicated in an independent validation dataset, involving the left inferior frontal triangular gyrus, bilateral thalamus, the left middle temporal lobe and the right superior temporal lobe.

For the primary dataset, a total of 5925 voxel-level functional connectivities had *p*-values smaller than the CDT *p* = 2 × 10^−8^ (*z* = 5.5) were identified, forming 327 FC clusters with spatial contiguity. The 33 largest FC clusters consisting of 4365 of the 5925 functional connectivities had cluster-size FWER-corrected *p* < 0.05, see Supplementary Table 2. Other small-sized FC clusters with cluster-size FWER-corrected *p* > 0.05 are identified as false positives and are filtered out. To show the 33 significant FC clusters clearly, each one of them was mapped to the corresponding region in AAL2^23^ atlas (i.e., the region comprising of the majority of the voxels), as shown in Supplementary Table 2. The anatomical location of the brain areas relating to the 33 FC clusters was shown in Fig. 1a and Supplementary Fig. 1. Meanwhile, to display the distribution of altered voxel-level functional connectivities within each AAL2 regions, the Manhattan plot of voxel-based BWAS results with voxels grouped in accordance with the AAL2 atlas labels was given as Supplementary Fig. 2.

The 33 significant FC clusters primarily involved thalamus, temporal cortex, inferior frontal cortex, precuneus, and subcortical structures like pallidum, see Fig. 1b. Among them, ten altered FC clusters involved bilateral thalamus, with the top 2 largest FC clusters connecting bilateral thalamus and middle/ superior temporal lobe. There were 5 FC clusters which connected the left inferior frontal gyrus (triangular and orbital part, including Broca’s area), to anterior cingulate cortex, left medial superior frontal gyrus, and subcortical regions (putamen and pallidum). In addition, there were 4 FC clusters which connected precuneus to right middle/superior temporal gyrus, hippocampus, and left calcarine, and 6 FC clusters connected pallidum to middle/superior temporal gyrus, the left orbital part of inferior frontal gyrus (IFG).

**Fig. 2:**
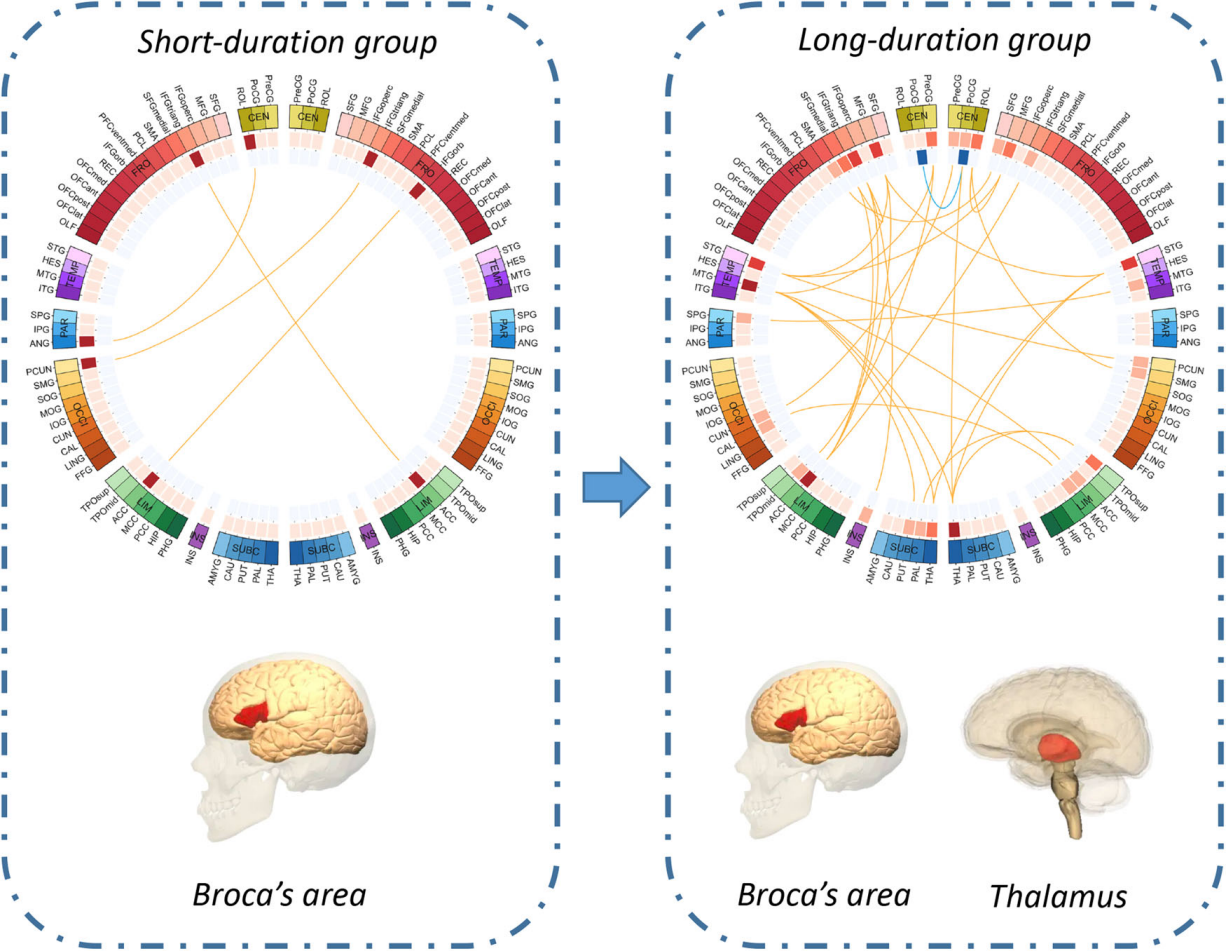
Significantly altered functional connectivity clusters (FC clusters) identified in both the short-duration group (11.2 ± 6.2 weeks) and the long-duration group (48.6 ± 22.8 weeks) first-episode schizophrenia (FES) patients. The color of the 3 circles (from inside to outside) denotes: the number of deceased functional connectivity; the number of increased functional connectivity; the 94 brain regions in automatic anatomical labeling (AAL2) atlas. Red lines mean that FES patients have a higher mean functional connectivity than healthy controls, and blue lines indicate the opposite. The anatomical abbreviations in the figure are shown in the Supplementary Table 1. Short-duration group had functional dysconnectivity mainly in the Broca’s area (the left inferior frontal gyrus), while long-duration group had widespread functional dysconnectivity involving both the left inferior frontal gyrus and the thalamus. The brain images in this figure are snapshots of 3D brain model of the Society for Neuroscience (2017) retrieved from https://www.brainfacts.org/3DBrain.

The results on the primary dataset were cross-validated on an independent dataset. 16 of the 33 FC clusters were significantly altered, see Fig. 2b and Supplementary Table 5, mostly involving left inferior frontal triangular gyrus, bilateral thalamus, left middle temporal lobe, and right superior temporal lobe.

### Thalamic and IFG connectivities are correlated with negative symptoms

We found that FC clusters between thalamus and superior/middle temporal gyrus, and between left inferior frontal gyrus (i.e., Broca’s area) and right anterior cingulate cortex were significantly correlated with PANSS negative total score, see Table 1. A further analysis of score of each item revealed that the connectivity between left inferior frontal gyrus (triangular and orbital part) with anterior cingulate cortex was correlated with conceptual disorganization (positive symptom), emotional withdrawal, passive/apathetic social withdrawal and difficulty in abstract thinking (negative symptom), which are related to language impairments, see Supplementary Table 6 and Supplementary Table 7.

**Table 1:** Correlations coefficient between the functional connectivity links and PANSS negative score.

Functional connectivity clusters				
Region 1	Region 2	*r*	*p*	FDR p
Left thalamus	Right superior temporal gyrus	0.3043	0.0005	0.0165
Right thalamus	Left middle temporal gyrus	0.2374	0.0060	0.0469
Right thalamus	Right superior temporal gyrus	0.2306	0.0063	0.0469
Left inferior frontal gyrus, triangular part	Right anterior cingulate cortex	0.2256	0.0068	0.0469
Right thalamus	Right superior temporal gyrus	0.2254	0.0071	0.0469
Left pallidum	Left middle temporal gyrus	0.2138	0.0129	0.0693
Right thalamus	Left middle temporal gyrus	0.2112	0.0149	0.0693
Right thalamus	Left middle temporal gyrus	0.2101	0.0168	0.0693
Left pallidum	Right superior temporal gyrus	0.2016	0.0191	0.0700
Left thalamus	Left middle temporal gyrus	0.1995	0.0235	0.0775
Left precuneus	Right superior temporal gyrus	0.1849	0.0324	0.0894
Left thalamus	Left middle temporal gyrus	0.1900	0.0325	0.0894

^a^*FDR* *p* False Discovery Rate corrected *p* value.

### IFG and thalamic dysconnectivity respectively in short and long duration FES

After identifying changes in FES patients overall, we then explored alterations specific to different stages, i.e., long (69 patients with a mean of 48.6 weeks) and short (69 patients with a mean of 11.2 weeks) duration group. We applied the cluster-level inference approach to identify significantly altered FC clusters (CDT *p* = 3 × 10^−7^ (*z* = 5) and cluster-size FWER-corrected *p* = 0.05) in long and short duration group. The results were shown in Supplementary Tables 3 and 4.

There were 4 FC clusters (Fig. 2, Supplementary Table 3) that can survive the cluster-size FWER-corrected *p* < 0.05 threshold in short-duration group including 3 FC clusters linking the left triangular inferior frontal gyrus, which is part of Broca’s area, the right orbital and triangular part of the inferior frontal gyrus to precuneus and anterior cingulate cortex in the opposite hemisphere. In the long-duration group, 29 FC clusters (Fig. 2, Supplementary Table 4) had cluster-size FWER-corrected *p* < 0.05 and most clusters linked thalamus bilaterally, middle temporal lobe and superior temporal lobe and triangular part of inferior frontal gyrus. There were 5 FC clusters involving thalamus in the top 10 clusters in long duration group.

### Language genes play more important roles in short-duration FES

**Fig. 3:**
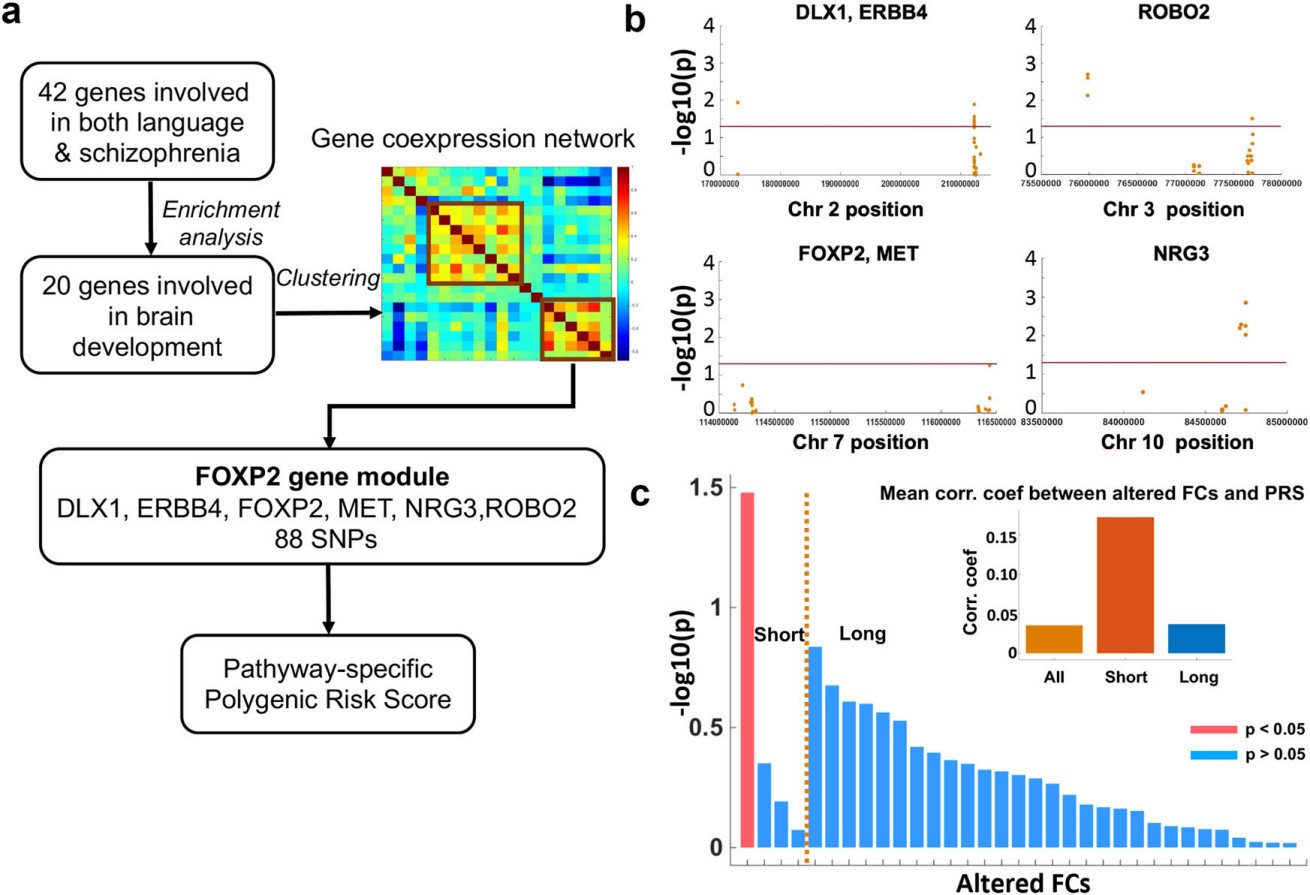
Genetic association analysis between altered functional connectivities (FCs) identified in FES patients and language-related genes. **a** Workflow to choose candidate genes in genetic association study. We identified one gene cluster (with 6 genes) containing FOXP2, which is related to neurodevelopment, language, and schizophrenia. **b** Association analysis between the 88 SNPs in the 6 genes in the FOXP2 cluster and schizophrenia. **c** Correlation coefficients between the pathway-specific polygenic risk scores (PRS) obtained from the above 6 genes and each of the altered FCs identified in both the short-duration patient group and the long-duration patient group. The bars represented the log *P* values of the correlation between the PRS and 4 altered FCs identified in short-duration group and the 29 FCs identified in the long-duration group (the red bar indicate *p* < 0.05). The upper right part of this figure showed the mean value of the correlation coefficients (between altered FCs and PRS) in different patients groups (orange for all FES patients, red for short-duration FES group, and blue for long-duration FES group).

To explore the genetic mechanism underlying the altered functional connectivity in FES patients, and specifically, the role of language-related genes regarding the language hypothesis, we performed genetic association analysis using pathway-specific polygenic risk scores obtained from the gene cluster involving FOXP2 (6 genes), which are implicated in both language and schizophrenia, and are associated with development, see Fig. 3a. First, we found that 20 SNPs in these genes are associated with disease (*p* < 0.05), see Fig. 3b. Then we calculated the correlation coefficients between the polygenic risk score from these 20 SNPs and the altered FC clusters identified in short-duration FES patients (4 FC clusters, see Supplementary Table 3) and long-duration FES patients (29 FC clusters, see Supplementary Table 4), respectively, and found that polygenic risk scores had stronger correlation coefficients (*t* (33) = 1.87, *p* = 0.036) with altered FC clusters identified in short-duration group (between left inferior frontal gyrus and right anterior cingulate cortex, and between left angular gyrus and left rolandic operculum), see Fig. 3c. This suggested that the 6 language genes together may be responsible for the FC changes in short-duration FES patients. In contrast, polygenic risk scores did not show significant correlation with any altered FC clusters identified in the longer duration group. To verify this we furthermore performed a comparison between the strength of correlation (a single correlation coefficient obtained by plotting the mean of all altered voxel-level FCs against polygenic risk score in each subject) in short duration (1375 voxel-level FCs) vs. long duration patient groups (13,233 voxel-level FCs). By non-parametric permutation (*n* = 10,000) we found a significant correlation between PRS and the mean FCs in the short-duration group (*p* = 0.049) but not the long-duration group (*p* = 0.716).

To establish of the specificity of the FOXP2 cluster genes in the functional connectivity abnormalities seen in our untreated schizophrenia samples, we furthermore reanalyzed genetic association using SNPs from all 20 genes that are not filtered by co-expression and FOXP2-linked clustering. The mean of the absolute correlation between the unfiltered PRS (from the 20 genes) and the altered FCs was lower than using the PRS obtained from the FOXP2 cluster genes, and there was no significant correlation between the unfiltered PRS and FCs in both the early and later stages of schizophrenia (mean correlation coefficient in patients of short and long illness duration being 0.069 and 0.062, respectively, without significant difference). This highlighted the specificity of FOXP2 gene assemblies with conjoint spatial expression in functional dysconnectivity seen in FES.

## Discussion

In a large sample of drug-naïve FES patients with a broad range of illness duration (1 to 100 weeks), we report that increased resting-state functional connectivity involving thalamus and Broca’s area as the predominant aberration in functional connectivity. Notably, for the first time we establish that irrespective of illness duration, untreated patients consistently show altered connectivity in inferior frontal gyrus (site of the Broca’s area) and superior/middle temporal gyrus, cortical regions critical for various levels of human language processing. For patients in the earliest stages of illness (shorter duration), mutations in the genetic determinants of language (FOXP2 related genes) play a critical role in the observed functional dysconnectivity between Broca’s area and anterior cingulate cortex. Finally, we also note that the degree of anterior cingulate cortex dysconnectivity with language-related areas (inferior frontal and superior temporal regions) is correlated with the severity of core negative symptoms of schizophrenia (emotional withdrawal, passive/apathetic social withdrawal and difficulty in abstract thinking), reflecting the primacy of language dysfunction in its pathophysiology.

Our results complement our previous findings of Broca’s area dysconnectivity in first-episode schizophrenia and thalamo-cortical dysconnectivity in chronic schizophrenia^8^. In contrast to our previous multi-site work, we used drug-naïve FES patients with wide distribution of illness duration (1 to 100 weeks) in the current study, allowing us to study the effect of chronicity in a drug-naïve state. The short-duration group in the current study had FC changes (mainly Broca’s area) similar to the first-episode group in our previous sample, while the long-duration group had FC changes (mainly thalamus and temporal lobe) similar to the chronic illness group in the previous sample. These complementary observations strongly suggest that Broca’s area (IFG) may be the ‘site of origin’ of FC changes in schizophrenia (see Fig. 2). This result is supported by recent findings of elevated PFC connectivity in FES patients^9^, and increased functional connectivity involving Broca’s area that could have prognostic and therapeutic implications^24^.

Broca’s area plays a key role in both syntactical and semantic processing of language. Structured language production plays an important role in multi-step thinking^25^, with aberrant connectivity of Broca’s region likely contributing to difficulties in negative symptoms and disorganization, often seen in early stages of psychosis, even before the onset of reality distortion^26^. Such symptoms are also of prognostic importance in the presence of psychosis^27^. In contrast, for patients with longer illness duration (23.3 weeks–100 weeks), the most significant changes involve the superior/middle temporal cortex and thalamus. The involvement of superior temporal cortex is suggestive of language-related processing deficits^28,29^, and can be viewed as an index of chronicity of the untreated illness. It is also possible that patients with predominant Broca’s area dysconnectivity present earlier due to higher illness severity or acuity. Longitudinal fMRI studies in untreated samples are required to test these speculations.

The changes in inferior frontal gyrus functional connectivity in short-duration patient group and its correlation with negative symptoms indicates that dysfunction in Broca’s area may be related to schizophrenia, in line with the hypothesis of Crow^15,16^. Crow, suggested that schizophrenia is the breakdown of the normal left-sided brain specialization for language and a “side effect” of human linguistic evolution^15,16^; this was supported by recent polygenic analysis of GWAS data^30^. On the basis of 42 candidate genes implicated in both language readiness and schizophrenia^31–33^, and clustered using their whole-brain gene expression profiles (Allen Brain Atlas), we identified a group of genes including FOXP2, which were closely associated with language, schizophrenia, and brain development. Association analysis between the 88 SNPs in the FOXP2 genes cluster and schizophrenia revealed IFG hyper-connectivity being closely related with polygenetic scores of the FOXP2 cluster in patients at an early-stage illness, while other genetic/non-genetic factors may influence the dysconnectivity patterns in those with longer duration of illness. This provides the first direct neuroimaging and genetic evidence for Crow’s linguistic primacy hypothesis. Our results also highlight the importance of teasing apart the effect of medication exposure and illness duration in imaging genetics studies of psychosis. We speculate that the genetic determinants of the initial functional dysconnectivity critical for symptom emergence are likely to be different from the determinants of illness persistence and chronicity. This is consistent with the observation that not all FES patients develop a persisting illness^6^, contributing to the heterogeneous trajectory of this condition. Compensatory brain changes may also occur after early psychotic symptoms^17^, contributing to structural or functional reorganization^34^, thus weakening the link between genetic risk factors and observed dysconnectivity phenotype.

Schizophrenia is considered by some authors as a ‘language-related human specific disease’ or ‘logopathy’, which promises a better understanding of the disease pathophysiology^35^. FOXP2 gene plays an important role in the development of the neural systems that mediate speech and language^36^. FOXP2 polymorphisms have been involved in neuropsychiatric and developmental disorders like schizophrenia^37,38^, autism spectrum disorders, and language disorder like dyslexia^39^. Importantly, FOXP2 polymorphisms have been shown to be associated with the severity of a core language symptom, i.e., formal thought disorder in schizophrenia^40^, and the inferior frontal gyrus is a site of pathology that has been most consistently associated with FOXP2 disruption in multiple studies^41^. In addition to speech and language disorders, FOXP2 has also been implicated it in adult ADHD^42,43^. A FOXP2 knockout models implicate a role for this gene in regulating dopamine in subcortical circuits^44^. Taken together, our results have the potential to tie up the linguistic basis of schizophrenia with its neurochemical basis in dopamine.

In our work, the increased functional connectivity of left IFG (see Supplementary Table 3), specifically with the opposite hemisphere, may be related to disrupted lateralization of schizophrenia patients, which may be associated with the language gene cluster that involves FOXP2, previously shown to be responsible for language lateralization in both healthy controls and FES patients^45,46^. Other genes in this FOXP2 gene cluster include NRG3, ERBB4, DLX1, ROBO2, and ROBO2, all are susceptibility gene in schizophrenia and is related to development^47^. Among them, the NRG3N, one in the large family of growth factors, and its receptors ERBB4 genes are part of the NRG-ERBB signaling network that has been implicated intensively in schizophrenia^48^. This pathway plays important roles in neural development including circuitry generation, axon ensheathment, neurotransmission, and synaptic plasticity. The variants of these genes have been shown to enhance synchronized oscillations of prefrontal cortex neurons via inhibitory synapses, possibly responsible for the increased functional connectivity related to IFG in first-episode patients^49^.

One limitation of our work is the lack of symptom scores or task parameters pertaining to linguistic deficits per se. So, we are not able to assess how language network dysconnectivity operates to influence the core feature of conceptual disorganisation in schizophrenia. We cannot interpret the dysconnectivity observed here as a substrate of the thought, language, and communication disorder in schizophrenia. We also caution readers against generalizing the reported observations to verbal performance and abilities in patients, both of which were not examined here.

A polygenic cluster related to language as well as schizophrenia, comprised of FOXP2, and NRG-ERBB signaling pathways, influences the connectivity of Broca’s area, which in turn influences the clinical expression of core symptoms in untreated early stages of schizophrenia. Our results, reported from a discovery as well as a validation sample of drug-naïve subjects, bridges the mechanistic gap between genetic variations and clinical symptoms of schizophrenia. It also raises the intriguing question of the role that linguistic interventions can play in reducing the pathophysiological processes that define early stages of schizophrenia.

## Methods

### Subjects

Resting-state fMRI and whole-exome sequencing data of all participants were collected at Shanghai Mental Health Center from 2016 to 2018. The study was approved by the Shanghai Mental Health Center Institutional Review Board, and all participants provided written informed consent. The discovery (primary) dataset included 138 patients and 112 controls (107 patients and 71 healthy controls with whole-exome sequencing data) and the validation dataset included 53 patients and 56 controls after excluding the subjects with mean framewise displacement exceeding 0.5 mm, see Table 2 for demographic and clinical data for details. All the subjects were Mandarin-speaking Han Chinese individuals from Shanghai metropolitan area. The FES patients were identified according to *DSM-IV* criteria^50^ by qualified psychiatrists using all available clinical information including a diagnostic interview of patients and their family, clinical case notes, and clinician’s observations. Symptom severity was measured using the standardized positive and negative syndrome scale (PANSS) assessment. FES patients were defined as having illness duration less than 2 years, as this is the most common definition used in the literature^51^. The duration of untreated illness [DUP] ranged from 1 to 100 weeks. There were 25 patients (18.1%) with illness duration longer than 52 weeks. It is important to note that this broad range of DUP (i.e., illness duration at the time of scanning) is representative of FES samples across the world, and in line with the findings that on average the DUP of lower and middle income countries is twice as long as the DUP in high-income countries^52^. All healthy subjects were assessed in accordance with *DSM-IV* criteria as being free of schizophrenia and other axis I disorders, and none had neurological diseases, head trauma, or substance abuse. All participants were between the ages of 14 and 45 years; were right-handed; had no history of substance abuse or suicidal ideation; and had no MRI contraindications.

**Table 2:** Demographic and clinical characteristics of first-episode schizophrenia patients and matched healthy controls in the primary and validation dataset.

	Primary dataset						Validation dataset			
Characteristic	FES	HC	Test statistic	*P* value	FES short group	FES long group	FES	HC	Test statistic	*P* value
Number of subjects	138	112	NA	NA	69	69	53	56	NA	NA
Age, mean (SD), years	24.0 (8.1)	24.5 (6.3)	−0.48	0.63	22.8 (7.8)	25.3(8.3)	25.5 (6.6)	25.8 (6.2)	−0.12	0.9
Gender (M/F)	79/ 59	50/ 62	3.92	0.05	45/ 24	34/ 35	31/ 22	30/ 26	0.41	0.68
Education level, mean (SD), years	11.6 (2.8)	12.8 (3.0)	−3.19	0.002	11.3 (2.8)	11.9 (2.8)	12.6 (3.2)	13.0 (2.7)	−0.08	0.78
Duration, weeks	29.9 (25.1)	NA	NA	NA	11.2 (6.2)	48.6 (22.8)	25.4 (18.1)	NA	NA	NA
PANSS positive score	20.5 (5.7)	NA	NA	NA	20.6 (5.8)	20.4 (5.6)	15.6 (4.4)	NA	NA	NA
PANSS negative score	14.9 (6.9)	NA	NA	NA	14.2 (6.5)	15.6 (7.2)	7.7 (2.9)	NA	NA	NA

^a^*PANSS* Positive and Negative Syndrome Scale.

^b^*FES* First Episode Schizophrenia group.

^c^*HC* Healthy Control group.

### Image acquisition and preprocessing

Resting-state fMRI data were acquired using a 3 T MRI scanner (Siemens) in an 8-min period in which the participants were awake in the scanner. A total of 240 volumes of images were obtained (TR/ TE: 2000/30 ms, Flip angle 77^。^matrix size: 64 × 64, voxel size: 3 × 3 × 3 mm^3^; FOV = 220 × 220 mm^2^, slices 50).

fMRI data were preprocessed using FSL^53^ and AFNI^54^. Images of each subject were corrected for slice timing (FSL) and motion with 24 head movement parameters^55^. We registered functional images to 3 mm standard Montreal Neurological Institute (MNI) space by firstly aligning functional images to the subject’s T1 structural images and then transformed them to the standard space (FSL). The functional volumes were spatially smoothed with a Gaussian kernel (6 mm^3^ full-width half-maximum (FWHM)). Wavelet despiking was applied to denoise the time series of voxels^56^, and we band-pass filtered the time series between 0.01 and 0.1 Hz using AFNI. White matter signal, cerebrospinal fluid signal, and global mean signal were also regressed out (AFNI) in line with our previous work^8,57^. Subjects with mean framewise displacement exceeding 0.5 mm were excluded from the analysis.

### Whole exome sequencing data preprocessing

Library preparation – The 300 ng genomic DNA were sheared with Covaris LE220 Sonicator (Covaris) to target of 150–200 bp average size. DNA libraries were prepared using SureSelectXT Human All Exon V6 + UTR (Agilent). The fragments were repaired the 3′ and 5′ overhangs using End repair mix (component of SureselectXT) and purified using Agencourt AMPure XP Beads (Beckman). Finally, the pre-capture libraries containing exome sequences were captured using SureSelect capture library kit (Agilent).

Illumina sequencing DNA concentration of the enriched sequencing libraries was measured with the Qubit 2.0 fluorometer dsDNA HS Assay (Thermo Fisher Scientific). Paired-end sequencing is performed using an Illumina NovaSeq6000 system following Illumina-provided protocols for 2 × 150 paired-end sequencing.

Variant Detection Gene variant refer to changes in the base of genome, including point mutations caused by single base changes, or deletions, duplications, or insertions of multiple bases. In this analysis, the workflow of Sentieon^58^ was carried out to detect SNV (Single Nucleotide Variants) and InDel (small insertions and deletions). The variant detection of analysis processes are as follows: 1. PF reads are aligned to the human reference genome(hg19) and sorted. 2. Mark duplicated PCR reads in bam files (Dedup.bam). 3. Reads in the region of InDel were realigned (InDel realign). 4. Base quality score recalibration (BQSR). 5. Detection of SNV and InDel. 6. Variant quality score recalibrate.

### Cluster-based brain-wide functional connectivity study

In the present study, we adopted a cluster-based brain-wide association study (BWAS) method^59^ to identify altered functional connectivity clusters in FES patients in the primary dataset. Each resting-state brain image included 47619 voxels (3 × 3 × 3 mm^3^) within the cerebrum (based on AAL2 atlas^23^, which was also used for labeling the clusters). For each participant, Pearson’s correlation coefficients between the fMRI time series at each brain voxel were computed followed by Fisher’s *z*-transformation. This resulted in a 47619 × 47619 functional connectivity matrix for each subject. A general linear model was used to compare functional connectivity between FES patients and healthy controls, with age, gender, education level, mean framewise displacement as covariates. A *z*-statistic was obtained which reflected the degree of change in patients for each voxel by voxel connectivity (i.e., each cell of the 47619 × 47619 matrix). A predefined cluster-defining threshold (CDT) of *p* = 2 × 10^−8^ (*z* = 5.5) was applied to each voxel-level test. Then the voxel–voxel connections with *p* value less than the CDT with edges linking a given pair of the same two distinct voxel clusters were identified as a functional connectivity cluster (FC cluster)^59^. To correct for multiple comparisons, the cluster-level inference method based on random field theory was used, which estimated the cluster-size family-wise error rate (FWER)-corrected *p* value using the size of each FC cluster, i.e., the number of voxel-level functional connectivities within it^59^. Large-sized FC clusters (FWER-corrected *p* < 0.05) were identified as significantly altered FC clusters in patients.

We then calculated the partial correlation coefficients between the strength of significantly altered FC clusters (FWER-corrected *p* < 0.05), specifically, the mean of all the voxel-level functional connectivities within it, with the positive and negative syndrome scale (PANSS) scores, with the same covariates as mentioned above. Here, 10,000 random permutations were implemented to estimate the null distribution of the correlation coefficients between functional connectivities and PANSS scores and also the statistical *p* values. A false discovery rate (FDR) procedure^60^ was used to correct for multiple hypothesis testing.

### Stage-specific changes of functional connectivity with illness duration

In order to evaluate the dynamic changes of functional connectivity with illness duration, we split the FES patients in the primary dataset into two groups based on the median of duration (23.3 weeks): short (69 patients with a mean of 11.2 weeks (1–22.3 weeks)) duration group and long (69 patients with a mean of 48.6 weeks (23.3–100 weeks)) duration group (Table 2), and compared with all the healthy controls (112 subjects), respectively, adopting cluster-based brain-wide functional connectivity analysis at the voxel-level. Due to the relatively smaller sample size, we adopted a correction threshold (CDT *p* = 3 × 10^−7^ (*z* = 5) and cluster-size FWER-corrected *p* = 0.05) in the cluster-level inference in the two groups.

### Cross-validation on an independent dataset

We validated our observations of the primary dataset in an independent validation dataset consisting of 109 participants (53 FES; mean age, 25.7 years; mean illness duration, 25.4 weeks). Specifically, we calculated the mean value of all the voxel-level functional connectivities within each FC cluster identified in the primary dataset in the independent validation dataset and then test whether these mean values were changed in the FES patients compared with healthy controls.

### Language dysfunction and negative symptoms

The dimension of disorganization and formal thought disorder correlates strongly with negative symptoms, as first shown by McGorry et al., in a factor analysis study^61^ (with predictive value of this combined dimension confirmed by Dominguez et al.^26^ in a 10-year longitudinal study). Furthermore a strong relationship exists between impoverishment (negative symptoms) and communication dysfunction in schizophrenia^62^, and this association occurs several years before positive psychotic symptoms appear^63^. Language dysfunction in schizophrenia is traditionally considered to include a positive dimension of incoherence, distractibility, and tangential speech and a negative dimension of reduced speech, poverty, and perseveration of ideas. Of these, the positive dimension is more episodic, seen during acute phases and share variance with other positive symptoms (delusions, hallucinations), while the negative dimension is more pervasive, trait like, and stable, making a suitable substrate for genetic studies^64^. Considering that the negative symptoms (Alogia, Autism, Ambivalence, and Affect Blunting) and disorganization (Loosening of Associations) are the fundamental features of this illness^65^, we consider the negative symptom dimension as an appropriate, stable proxy for the clinical expression of impoverished thought, language, and communication in schizophrenia.

### Genetic association analysis

To test the hypothesis if there is a relation between the altered functional connectivity in language-related areas and language-related gene mutations, we carried out a genetic association analysis using the pathway-based polygenic risk scores^18,19^, as gene sets centering on putative core function, or belonging to specific biologically relevant pathways, may make a larger-than-expected contribution to polygenic risk^21,66^. In particular, we chose the candidate genes that are related to schizophrenia, development, and language by combing knowledge from multiple sources, including existing literature, gene ontology analysis, and brain gene expression data. These genes involved the Forkhead box P2 (FOXP2) gene that is well-known to be associated with language which is shown to be a promising candidate in language dysfunction in schizophrenia^47^.

The workflow of the genetic analysis was shown in Fig. 3a. We started from the 42 genes that are shown to be related to both language readiness^31–33^ and schizophrenia listed by Murphy & Benitez-Burraco in an extensive recent review^47^. We performed functional enrichment analysis (by ToppGene Suite^67^) and selected 20 genes that had an established ontological role in the human brain development, in line with the neurodevelopmental hypothesis of schizophrenia^68^. Next, we clustered these 20 genes using their whole-brain gene expression profiles (obtained from six adult brains in the Allen Brain Atlas^69^) by K-means algorithm, and we identified the genes that are co-expressed with FOXP2, which we termed as the FOXP2 cluster genes^70^. We chose to focus on FOXP2 gene cluster given the extant evidence that this gene bridges neural plasticity and spoken language^71,72^, and its expression is enriched in language areas and it plays a role in their specialization^73^. The clustering step ensured that we identified functional gene assemblies that require a co-regulated transcriptional profile and conjoint spatial expression to influence language function^74^. Thus, we identified a distinct module of language, brain development and schizophrenia related, co-expressed ensemble of genes not only important for brain maturation in anatomically modern humans, but also relevant to the expression of the clinical and connectivity phenotype of schizophrenia^47^.

We finally calculated the polygenic risk score using the SNPs in this FOXp2 gene cluster (*p* < 0.05), i.e., weighted sum of these SNPs with log (odds ratio) for each SNP being the weight. The strength of the altered FC clusters that are identified in the whole FES group, long-duration patients group, and short-duration patients group were respectively extracted in all 107 patients with both genetic and imaging data in the whole FES group. Then we explored the relationship between this pathway-specific polygenic risk score and strength of the FC clusters in all 107 patients. Our general goal was to identify the correlation between schizophrenia-related network-level dysconnectivity and genetic mutations in language-readiness genes, rather than to predict disease state using all genotype data as traditional GWAS study does. Therefore we used the SNP variants called from exome sequencing data (specifically the FOXP2 gene cluster exomic loci) for the schizophrenia samples, following the principle of pathway-specific PRS. Note that exome data has been frequently used in imaging-genetic studies in psychiatric disorders^75,76^.

### Reporting summary

Further information on research design is available in the Nature Research Reporting Summary linked to this article.

## Supplementary information

Supplementary Information

reporting summary

## Data Availability

The datasets generated during the current study are not publicly available due to ethical codes for this study but are available from the corresponding author on reasonable request with the approval of The Research Ethics Committee of the Shanghai Jiao Tong University School of Medicine.

## Code availability

The BWAS: Voxel-level connectome-wide association studies code is publicly available at https://github.com/weikanggong/BWAS. The code used for these analyses is publicly available at the following url: https://osf.io/2zaqv/.
