# Supplementary material for: The genetic determinants of language network dysconnectivity in drug-naïve early stage schizophrenia
Source: NPJ Schizophr. 2021 Mar 3;7:18. doi: 10.1038/s41537-021-00141-8 (PMC7930279; doi:10.1038/s41537-021-00141-8)
Supplement: Supplementary file 1 — Supplementary Information [file 41537_2021_141_MOESM1_ESM.pdf]

Supplementary Figure 1. Voxels showing the largest number of brain-wide voxel-level functional connectivity differences in drug-naïve first-episode schizophrenia patients (in the primary dataset involving 138 patients) compared to matched controls, which were identified by voxel-based BWAS. The CDT is p=2×10-8 (z=5.5) and cluster-size FWER p threshold is 0.05. The color bar represents the measure of association (MA) which is the number of significantly altered functional connectivities relating to each voxel.

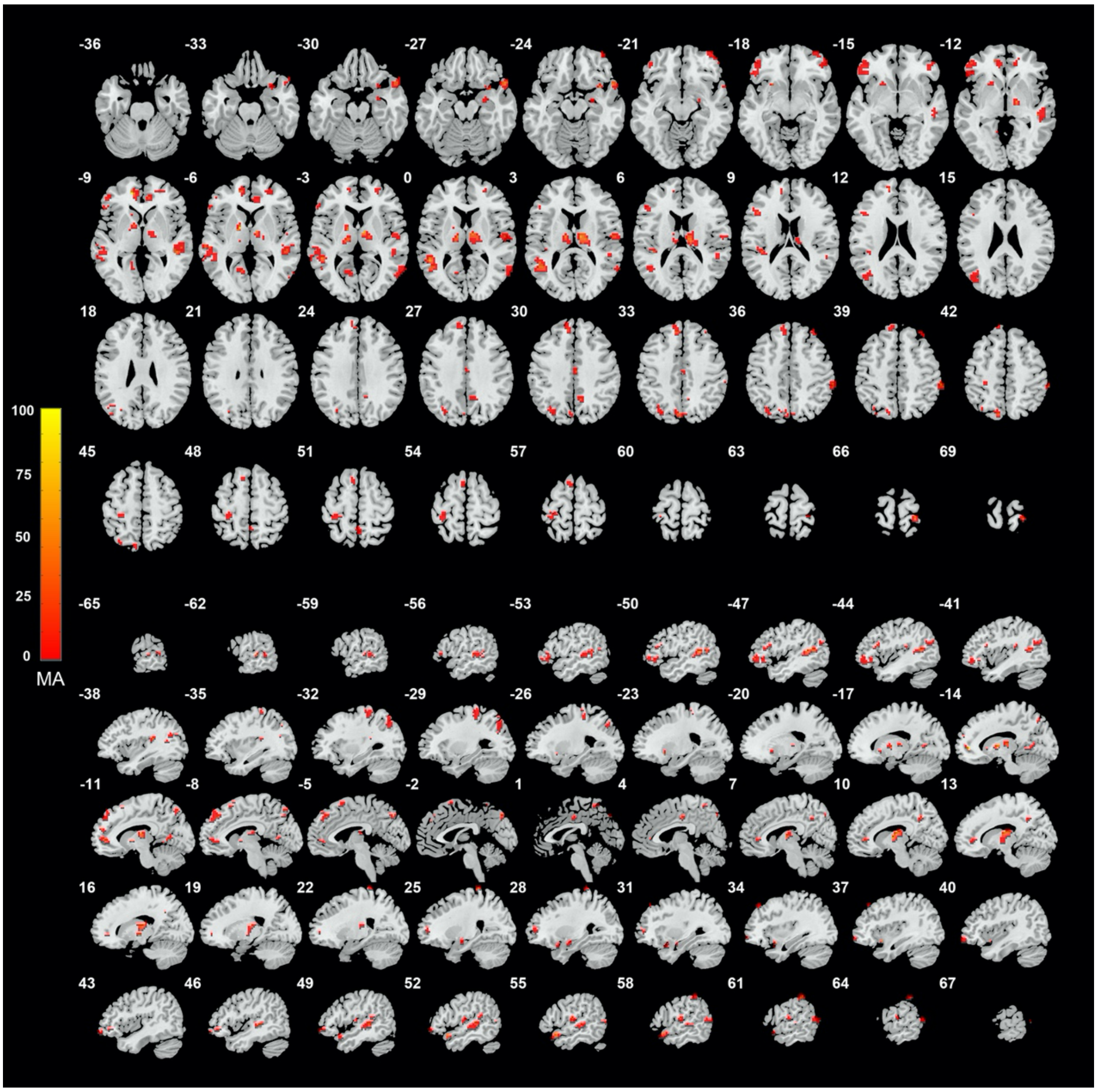

Supplementary Figure 2. Manhattan plot of voxel-based BWAS results with voxels grouped in accordance with the AAL2 atlas labels ($p < 1\times10^{-4}$). Each point represents a functional connectivity and the order of the bars is shown in Table S1. The width of each bar reflects the number of voxels in each AAL2 brain area. IFGtriang = inferior frontal gyrus, triangular part; ACC = anterior cingulate cortex; PreCG = precentral gyrus; PAL = pallidum; THA = thalamus; TPOsup = Temporal pole: superior temporal gyrus. TPOmid = Temporal pole: middle temporal gyrus.

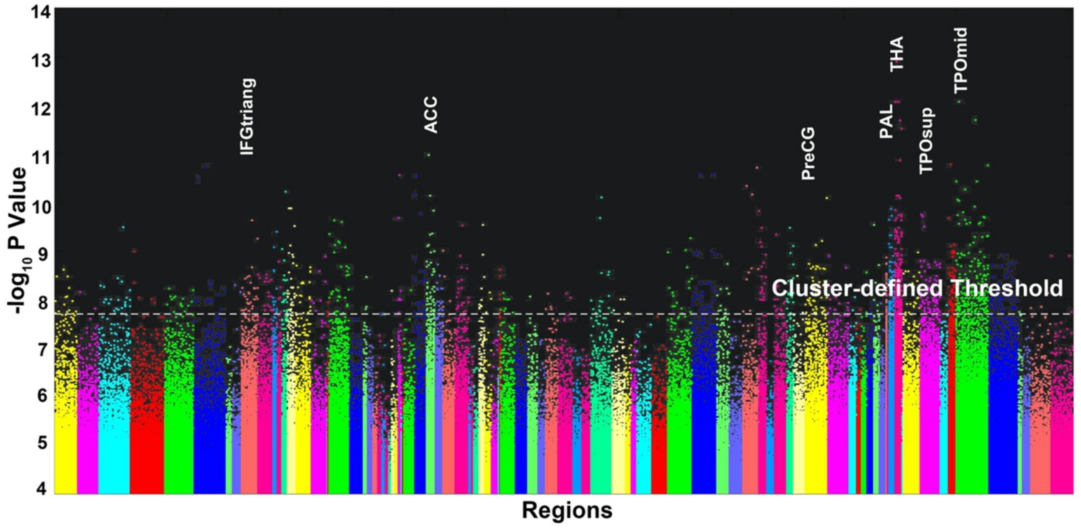

Supplementary Table 1. The anatomical regions defined in each hemisphere and their labels in the automated anatomical labeling atlas AAL2[1]. Column 4 provides a set of possible abbreviations for the anatomical descriptions.

NO.	ANATOMICAL DESCRIPTION	LABEL aal2.nii.gz	POSSIBLE ABBREVIATION
1,2	Precentral gyrus	Precentral	PreCG
3, 4	Superior frontal gyrus, dorsolateral	Frontal_Sup	SFG
5, 6	Middle frontal gyrus	Frontal_Mid	MFG
7, 8	Inferior frontal gyrus, opercular part	Frontal_Inf_Oper	IFGoperc
9, 10	Inferior frontal gyrus, triangular part	Frontal_Inf_Tri	IFGtriang
11, 12	IFG pars orbitalis,	Frontal_Inf_Orb	IFGorb
13, 14	Rolandic operculum	Rolandic_Oper	ROL
15, 16	Supplementary motor area	Supp_Motor_Area	SMA
17, 18	Olfactory cortex	Olfactory	OLF
19, 20	Superior frontal gyrus, medial	Frontal_Sup_Med	SFGmedial
21, 22	Superior frontal gyrus, medial orbital	Frontal_Med_Orb	PFCventmed
23, 24	Gyrus rectus	Rectus	REC
25, 26	Medial orbital gyrus	OFCmed	OFCmed
27, 28	Anterior orbital gyrus	OFCant	OFCant
29, 30	Posterior orbital gyrus	OFCpost	OFCpost
31, 32	Lateral orbital gyrus	OFClat	OFClat
33, 34	Insula	Insula	INS
35, 36	Anterior cingulate & paracingulate gyri	Cingulate_Ant	ACC
37, 38	Middle cingulate & paracingulate gyri	Cingulate_Mid	MCC
39, 40	Posterior cingulate gyrus	Cingulate_Post	PCC
41, 42	Hippocampus	Hippocampus	HIP
43, 44	Parahippocampal gyrus	ParaHippocampal	PHG
45, 46	Amygdala	Amygdala	AMYG
47, 48	Calcarine fissure and surrounding cortex	Calcarine	CAL
49, 50	Cuneus	Cuneus	CUN
51, 52	Lingual gyrus	Lingual	LING
53, 54	Superior occipital gyrus	Occipital_Sup	SOG
55, 56	Middle occipital gyrus	Occipital_Mid	MOG
57, 58	Inferior occipital gyrus	Occipital_Inf	IOG
59, 60	Fusiform gyrus	Fusiform	FFG
61, 62	Postcentral gyrus	Postcentral	PoCG
63, 64	Superior parietal gyrus	Parietal_Sup	SPG
65, 66	Inferior parietal gyrus, excluding supramarginal and angular gyri	Parietal_Inf	IPG
67, 68	SupraMarginal gyrus	SupraMarginal	SMG
69, 70	Angular gyrus	Angular	ANG
71, 72	Precuneus	Precuneus	PCUN
73, 74	Paracentral lobule	Paracentral_Lobule	PCL
75, 76	Caudate nucleus	Caudate	CAU
77, 78	Lenticular nucleus, Putamen	Putamen	PUT
79, 80	Lenticular nucleus, Pallidum	Pallidum	PAL
81, 82	Thalamus	Thalamus	THA
83, 84	Heschl’s gyrus	Heschl	HES
85, 86	Superior temporal gyrus	Temporal_Sup	STG
87, 88	Temporal pole: superior temporal gyrus	Temporal_Pole_Sup	TPOsup
89, 90	Middle temporal gyrus	Temporal_Mid	MTG
91, 92	Temporal pole: middle temporal gyrus	Temporal_Pole_Mid	TPOmid
93, 94	Inferior temporal gyrus	Temporal_Inf	ITG

Supplementary Table 2. Altered functional connectivity clusters identified in all the 138 first-episode schizophrenia patients (33 FC clusters, CDT Z<5.5). "increased" means FC of patient group is increased compared to controls.

Functional connectivity clusters		Direction	Cluster size	P-value	FWER p-value	Number of voxels in region 1	Number of voxels in region 2
'Thalamus_R'	'Temporal_Mid_L'	increased	899	1.10E-07	1.80E-06	81	98
'Thalamus_R'	'Temporal_Pole_Sup_R'	increased	428	3.68E-06	6.05E-05	55	38
'Parietal_Inf_R'	'Rolandic_Oper_R'	increased	262	2.43E-05	3.99E-04	26	41
'Thalamus_L'	'Temporal_Mid_L'	increased	222	4.31E-05	7.07E-04	28	47
'Frontal_Sup_Medial_L'	'Cingulate_Ant_L'	increased	190	7.16E-05	1.17E-03	56	29
'Thalamus_R'	'Temporal_Sup_R'	increased	164	1.13E-04	1.85E-03	37	46
'Postcentral_R'	'Postcentral_L'	decreased	161	1.20E-04	1.96E-03	17	51
'Angular_L'	'Rolandic_Oper_L'	increased	149	1.50E-04	2.47E-03	37	19
'Precuneus_L'	'Temporal_Mid_R'	increased	146	1.60E-04	2.62E-03	18	37
'Cingulate_Ant_R'	'Frontal_Inf_Tri_L'	increased	144	1.66E-04	2.73E-03	22	36
'Precuneus_L'	'Temporal_Sup_R'	increased	139	1.84E-04	3.02E-03	25	39
'Temporal_Sup_R'	'Thalamus_R'	increased	134	2.04E-04	3.35E-03	34	15
'Thalamus_L'	'Temporal_Sup_R'	increased	124	2.54E-04	4.16E-03	22	25
'Parietal_Inf_L'	'Insula_R'	increased	99	4.62E-04	7.55E-03	45	9
'Frontal_Inf_Tri_L'	'Frontal_Sup_Medial_L'	increased	98	4.74E-04	7.75E-03	26	20
'Precuneus_R'	'Calcarine_L'	increased	91	5.71E-04	9.34E-03	18	26
'Cingulate_Ant_L'	'Frontal_Inf_Orb_2_L'	increased	82	7.37E-04	1.20E-02	12	40
'Cingulate_Mid_R'	'Frontal_Mid_2_R'	increased	67	1.18E-03	1.92E-02	12	24
'Pallidum_L'	'Temporal_Sup_R'	increased	65	1.26E-03	2.05E-02	6	39
'Temporal_Mid_L'	'Thalamus_R'	increased	62	1.40E-03	2.27E-02	15	17
'Temporal_Sup_R'	'Thalamus_R'	increased	60	1.50E-03	2.44E-02	12	18
'Precuneus_L'	'Hippocampus_R'	increased	57	1.68E-03	2.72E-02	11	15
'Temporal_Mid_L'	'Thalamus_R'	increased	57	1.68E-03	2.72E-02	14	14
'Frontal_Sup_2_L'	'Frontal_Sup_2_R'	increased	56	1.74E-03	2.82E-02	8	24
'Pallidum_L'	'Temporal_Pole_Sup_R'	increased	51	2.12E-03	3.42E-02	19	11
'Pallidum_L'	'Temporal_Mid_L'	increased	49	2.30E-03	3.70E-02	6	26
'Supp_Motor_Area_L'	'Pallidum_L'	increased	48	2.40E-03	3.86E-02	23	7
'Pallidum_L'	'Frontal_Inf_Orb_2_L'	increased	46	2.61E-03	4.19E-02	8	15
'Cingulate_Ant_L'	'Frontal_Inf_Orb_2_R'	increased	44	2.85E-03	4.57E-02	7	21
'Frontal_Mid_2_R'	'OFCpost_R'	increased	43	2.98E-03	4.77E-02	11	9
'Frontal_Inf_Tri_L'	'Putamen_L'	increased	43	2.98E-03	4.77E-02	31	12
'Temporal_Mid_L'	'Pallidum_L'	increased	43	2.98E-03	4.77E-02	19	10
'Temporal_Mid_L'	'Thalamus_L'	increased	42	3.12E-03	4.99E-02	16	7

Supplementary Table 3. Altered functional connectivity clusters identified in the 69 short-duration first-episode schizophrenia patients (4 FC clusters in this group, CDT Z<5). “increased” means FC of patient group is increased compared to controls.

Functional connectivity clusters		Cluster size	Direction	P-value	FWER p-value	Number of voxels in region 1	Number of voxels in region 2
'Angular_L'	'Rolandic_Oper_L'	506	increased	2.53E-05	3.21E-03	64	43
'Frontal_Inf_Tri_R'	'Precuneus_L'	346	increased	8.92E-05	1.13E-02	73	42
'Frontal_Inf_Orb_2_R'	'Cingulate_Ant_L'	305	increased	1.31E-04	1.65E-02	33	70
'Frontal_Inf_Tri_L'	'Cingulate_Ant_R'	218	increased	3.38E-04	4.19E-02	56	36

Supplementary Table 4. Altered functional connectivity clusters identified in the 69 long-duration first-episode schizophrenia patients (29 FC clusters in this group, CDT Z<5). “increased” means FC of patient group is increased compared to controls.

**Functional connectivity clusters**		**Cluster size**	**Direction**	**P-value**	**FWER p-value**	**Number of voxels in region 1**	**Number of voxels in region 2**
Frontal_Mid_2_R'	'Temporal_Sup_L'	1207	increased	5.75E-07	7.65E-05	117	159
'Thalamus_R'	'Temporal_Mid_L'	1030	increased	1.20E-06	1.60E-04	81	139
'Frontal_Mid_2_L'	'Temporal_Sup_R'	900	increased	2.19E-06	2.92E-04	69	119
'SupraMarginal_R'	'Rolandic_Oper_R'	786	increased	3.90E-06	5.19E-04	57	98
'Temporal_Mid_L'	'Putamen_L'	724	increased	5.46E-06	7.27E-04	100	111
'Thalamus_R'	'Postcentral_R'	607	increased	1.09E-05	1.45E-03	75	57
'Thalamus_R'	'Temporal_Pole_Sup_R'	592	increased	1.20E-05	1.59E-03	58	64
'Frontal_Sup_Medial_L'	'Cingulate_Ant_L'	592	increased	1.20E-05	1.59E-03	104	51
'Thalamus_L'	'Temporal_Sup_R'	577	increased	1.32E-05	1.76E-03	46	65
'Thalamus_L'	'Temporal_Mid_L'	558	increased	1.50E-05	1.99E-03	57	104
'Frontal_Mid_2_L'	'Cingulate_Ant_L'	419	increased	4.11E-05	5.46E-03	86	28
'Precuneus_R'	'Temporal_Sup_L'	382	increased	5.59E-05	7.41E-03	81	51
'Frontal_Mid_2_R'	'Rolandic_Oper_R'	380	increased	5.69E-05	7.54E-03	83	59
'Frontal_Sup_2_R'	'Supp_Motor_Area_L'	365	increased	6.48E-05	8.59E-03	41	48
'Cingulate_Ant_R'	'Temporal_Mid_L'	346	increased	7.68E-05	1.02E-02	81	52
'Frontal_Mid_2_L'	'Temporal_Sup_L'	343	increased	7.89E-05	1.05E-02	43	56
'Postcentral_L'	'Postcentral_R'	342	decreased	7.97E-05	1.06E-02	56	35
'Putamen_L'	'Temporal_Mid_L'	332	increased	8.74E-05	1.16E-02	59	82
'Precentral_R'	'Cuneus_L'	313	increased	1.05E-04	1.39E-02	25	87
'Cingulate_Mid_R'	'Calcarine_L'	305	increased	1.13E-04	1.50E-02	25	50
'Precentral_L'	'Temporal_Pole_Mid_L'	276	increased	1.53E-04	2.01E-02	31	54
'Frontal_Inf_Tri_L'	'Cingulate_Ant_L'	276	increased	1.53E-04	2.01E-02	42	55
'Frontal_Inf_Tri_L'	'Insula_L'	270	increased	1.63E-04	2.14E-02	59	36
'Thalamus_R'	'Temporal_Sup_R'	237	increased	2.36E-04	3.09E-02	49	60
'Precentral_L'	'Temporal_Mid_L'	220	increased	2.90E-04	3.78E-02	20	58
'Parietal_Sup_L'	'Temporal_Mid_R'	220	increased	2.90E-04	3.78E-02	37	48
'Frontal_Inf_Tri_R'	'Cingulate_Ant_L'	215	increased	3.08E-04	4.02E-02	38	40
'Pallidum_L'	'Temporal_Pole_Sup_R'	213	increased	3.16E-04	4.12E-02	60	24
'Frontal_Inf_Tri_L'	'Frontal_Sup_Medial_L'	206	increased	3.46E-04	4.50E-02	44	14

Supplementary Table 5. Cross-validated functional connectivity clusters in the independent validation dataset (53 patients and 56 healthy controls).

**Functional connectivity clusters**		**P-value**	**T-value**
'Thalamus_R'	'Temporal_Mid_L'	0.035	2.135
'Thalamus_R'	'Temporal_Pole_Sup_R'	0.001	4.333
'Parietal_Inf_R'	'Rolandic_Oper_R'	0.048	2.000
'Thalamus_L'	'Temporal_Mid_L'	0.014	2.511
'Thalamus_R'	'Temporal_Sup_R'	0.000	4.166
'Precuneus_L'	'Temporal_Mid_R'	0.040	2.083
'Cingulate_Ant_R'	'Frontal_Inf_Tri_L'	0.047	2.014
'Temporal_Sup_R'	'Thalamus_R'	0.001	3.456
'Thalamus_L'	'Temporal_Sup_R'	0.001	4.360
'Pallidum_L'	'Temporal_Sup_R'	0.004	2.926
'Temporal_Sup_R'	'Thalamus_R'	0.001	4.174
'Pallidum_L'	'Temporal_Pole_Sup_R'	0.003	3.087
'Pallidum_L'	'Temporal_Mid_L'	0.017	2.434
'Supp_Motor_Area_L'	'Pallidum_L'	0.005	2.846
'Cingulate_Ant_L'	'Frontal_Inf_Orb_2_R'	0.001	3.276
'Temporal_Mid_L'	'Pallidum_L'	0.031	2.189

Supplementary Table 6. Correlations between the functional connectivity clusters and PANSS negative scores in all patients. (p values)

Functional connectivity clusters		S1	S2	S3	S4	S5	S6	S7
'Thalamus_R'	'Temporal_Mid_L'	0.112	0.011	0.010	0.100	0.069	0.422	0.074
'Thalamus_R'	'Temporal_Pole_Sup_R'	0.423	0.689	0.842	0.924	0.685	0.532	0.806
'Parietal_Inf_R'	'Rolandic_Oper_R'	0.631	0.762	0.436	0.823	0.334	0.401	0.674
'Thalamus_L'	'Temporal_Mid_L'	0.202	0.085	0.020	0.227	0.063	0.187	0.005
'Frontal_Sup_Medial_L'	'Cingulate_Ant_L'	0.111	0.139	0.124	0.154	0.086	0.478	0.130
'Thalamus_R'	'Temporal_Sup_R'	0.008	0.007	0.012	0.040	0.098	0.774	0.015
'Postcentral_R'	'Postcentral_L'	0.752	0.392	0.879	0.769	0.755	0.769	0.678
'Angular_L'	'Rolandic_Oper_L'	0.279	0.250	0.031	0.033	0.350	0.781	0.834
'Precuneus_L'	'Temporal_Mid_R'	0.787	0.877	0.467	0.876	0.347	0.786	0.566
'Cingulate_Ant_R'	'Frontal_Inf_Tri_L'	0.052	0.019	0.280	0.009	0.036	0.087	0.054
'Precuneus_L'	'Temporal_Sup_R'	0.018	0.039	0.180	0.050	0.225	0.207	0.491
'Temporal_Sup_R'	'Thalamus_R'	0.012	0.020	0.069	0.275	0.130	0.835	0.094
'Thalamus_L'	'Temporal_Sup_R'	0.005	0.002	0.005	0.013	0.003	0.225	0.001
'Parietal_Inf_L'	'Insula_R'	0.747	0.356	0.666	0.420	0.722	0.818	0.018
'Frontal_Inf_Tri_L'	'Frontal_Sup_Medial_L'	0.389	0.680	0.362	0.373	0.546	0.242	0.789
'Precuneus_R'	'Calcarine_L'	0.744	0.371	0.170	0.979	0.197	0.759	0.322
'Cingulate_Ant_L'	'Frontal_Inf_Orb_2_L'	0.283	0.386	0.086	0.067	0.747	0.349	0.250
'Cingulate_Mid_R'	'Frontal_Mid_2_R'	0.319	0.916	0.598	0.127	0.423	0.989	0.425
'Pallidum_L'	'Temporal_Sup_R'	0.055	0.155	0.013	0.167	0.011	0.396	0.009
'Temporal_Mid_L'	'Thalamus_R'	0.046	0.016	0.034	0.148	0.103	0.058	0.197
'Temporal_Sup_R'	'Thalamus_R'	0.004	0.006	0.059	0.106	0.373	0.050	0.079
'Precuneus_L'	'Hippocampus_R'	0.931	0.771	0.822	0.388	0.772	0.498	0.097
'Temporal_Mid_L'	'Thalamus_R'	0.022	0.014	0.004	0.195	0.049	0.342	0.024
'Frontal_Sup_2_L'	'Frontal_Sup_2_R'	0.306	0.466	0.108	0.173	0.106	0.796	0.067
'Pallidum_L'	'Temporal_Pole_Sup_R'	0.938	0.782	0.096	0.335	0.015	0.438	0.171
'Pallidum_L'	'Temporal_Mid_L'	0.057	0.040	0.046	0.131	0.025	0.166	0.016
'Supp_Motor_Area_L'	'Pallidum_L'	0.456	0.106	0.099	0.303	0.677	0.291	0.037
'Pallidum_L'	'Frontal_Inf_Orb_2_L'	0.268	0.433	0.184	0.025	0.386	0.930	0.114
'Cingulate_Ant_L'	'Frontal_Inf_Orb_2_R'	0.989	0.725	0.814	0.648	0.269	0.873	0.321
'Frontal_Mid_2_R'	'OFCpost_R'	0.962	0.915	0.747	0.756	0.515	0.931	0.570
'Frontal_Inf_Tri_L'	'Putamen_L'	0.114	0.120	0.282	0.494	0.193	0.648	0.168
'Temporal_Mid_L'	'Pallidum_L'	0.084	0.126	0.208	0.745	0.158	0.461	0.015
'Temporal_Mid_L'	'Thalamus_L'	0.170	0.176	0.084	0.362	0.018	0.120	0.003

[a]S1: Blunted affect; S2: Emotional withdrawal; S3: Poor rapport; S4: Passive/apathetic social; withdrawal; S5: Difficulty in abstract thinking; S6: Lack of spontaneity and flow of conversation; S7: Stereotyped thinking

Supplementary Table 7. Correlations between the functional connectivity clusters and PANSS positive scores in all the 138 patients. (p values)

**Functional connectivity clusters**		**S1**	**S2**	**S3**	**S4**	**S5**	**S6**	**S7**
'Thalamus_R'	'Temporal_Mid_L'	0.829	0.265	0.625	0.870	0.936	0.501	0.432
'Thalamus_R'	'Temporal_Pole_Sup_R'	0.370	0.927	0.311	0.969	0.200	0.178	0.329
'Parietal_Inf_R'	'Rolandic_Oper_R'	0.268	0.489	0.837	0.374	0.560	0.918	0.316
'Thalamus_L'	'Temporal_Mid_L'	0.768	0.045	0.423	0.161	0.821	0.125	0.421
'Frontal_Sup_Medial_L'	'Cingulate_Ant_L'	0.867	0.201	0.462	0.810	0.023	0.628	0.991
'Thalamus_R'	'Temporal_Sup_R'	0.706	0.181	0.745	0.939	0.165	0.701	0.431
'Postcentral_R'	'Postcentral_L'	0.917	0.530	0.693	0.858	0.461	0.586	0.800
'Angular_L'	'Rolandic_Oper_L'	0.796	0.601	0.678	0.694	0.037	0.515	0.017
'Precuneus_L'	'Temporal_Mid_R'	0.777	0.462	0.592	0.583	0.606	0.663	0.456
'Cingulate_Ant_R'	'Frontal_Inf_Tri_L'	0.408	0.399	0.726	0.495	0.096	0.777	0.906
'Precuneus_L'	'Temporal_Sup_R'	0.790	0.971	0.544	0.021	0.140	0.506	0.445
'Temporal_Sup_R'	'Thalamus_R'	0.354	0.565	0.552	0.877	0.279	0.784	0.171
'Thalamus_L'	'Temporal_Sup_R'	0.946	0.048	0.741	0.262	0.838	0.828	0.006
'Parietal_Inf_L'	'Insula_R'	0.048	0.744	0.022	0.417	0.855	0.300	0.551
'Frontal_Inf_Tri_L'	'Frontal_Sup_Medial_L'	0.766	0.366	0.284	0.789	0.190	0.917	0.346
'Precuneus_R'	'Calcarine_L'	0.209	0.106	0.260	0.740	0.729	0.667	0.779
'Cingulate_Ant_L'	'Frontal_Inf_Orb_2_L'	0.978	0.660	0.155	0.208	0.067	0.858	0.878
'Cingulate_Mid_R'	'Frontal_Mid_2_R'	0.660	0.975	0.859	0.681	0.669	0.595	0.729
'Pallidum_L'	'Temporal_Sup_R'	0.602	0.004	0.503	0.055	0.044	0.534	0.146
'Temporal_Mid_L'	'Thalamus_R'	0.942	0.355	0.803	0.777	0.117	0.914	0.577
'Temporal_Sup_R'	'Thalamus_R'	0.091	0.945	0.051	0.465	0.356	0.004	0.744
'Precuneus_L'	'Hippocampus_R'	0.336	0.879	0.315	0.591	0.798	0.688	0.797
'Temporal_Mid_L'	'Thalamus_R'	0.765	0.235	0.640	0.603	0.711	0.185	0.778
'Frontal_Sup_2_L'	'Frontal_Sup_2_R'	0.992	0.037	0.594	0.343	0.445	0.838	0.722
'Pallidum_L'	'Temporal_Pole_Sup_R'	0.465	0.008	0.923	0.090	0.323	0.459	0.613
'Pallidum_L'	'Temporal_Mid_L'	0.423	0.054	0.074	0.175	0.846	0.964	0.242
'Supp_Motor_Area_L'	'Pallidum_L'	0.063	0.002	0.645	0.015	0.046	0.057	0.004
'Pallidum_L'	'Frontal_Inf_Orb_2_L'	0.406	0.012	0.789	0.068	0.472	0.718	0.294
'Cingulate_Ant_L'	'Frontal_Inf_Orb_2_R'	0.995	0.690	0.413	0.074	0.109	0.653	0.627
'Frontal_Mid_2_R'	'OFCpost_R'	0.987	0.781	0.357	0.986	0.993	0.077	0.297
'Frontal_Inf_Tri_L'	'Putamen_L'	0.814	0.177	0.834	0.925	0.846	0.384	0.248
'Temporal_Mid_L'	'Pallidum_L'	0.205	0.129	0.117	0.381	0.947	0.820	0.504
'Temporal_Mid_L'	'Thalamus_L'	0.841	0.029	0.828	0.328	0.722	0.074	0.468

[a]S1: Delusions; S2: Conceptual disorganization; S3: Hallucinations; S4: Excitement; S5: Grandiosity; S6: Suspiciousness/persecution; S7: Hostility

Supplementary Note 1

Currently, there is no consensus in the neuroimaging field whether to remove global signal when computing functional connectivity. Global signal removal has been shown to reduce physiological noise and movement-related effects [2], thus improving its reliability [2-4], although it can increase the number of negative functional connectivities [5]. The major argument against global signal removal is the introduction of spurious correlations. In our study, we are interested in only the difference between control and patient groups in terms of their functional connectivity strength, irrespective of the sign of the functional connectivity. Global signals in both groups are regressed using an identical approach thus reducing the possibility of introducing spurious group differences. Therefore, we believe that removing global signals will have limited effect on the statistical test between two groups. Moreover, though global signal removal can increase the frequency of pairwise negative correlation coefficients, we consider negative correlation only on relative terms rather than anticorrelations, which is consistent with Murphy et. [6].

Though there is still controversy about global signal removal in group-wise analysis [7], the key functional connectivity changes we identified are clearly consistent among different datasets belonging to the same illness stage. Future investigations are needed to access systematically the impact of global signal.

Supplementary Reference

1 Rolls, E. T., Joliot, M. & Tzourio-Mazoyer, N. Implementation of a new parcellation of the orbitofrontal cortex in the automated anatomical labeling atlas. *NeuroImage* **122**, 1-5, doi:10.1016/j.neuroimage.2015.07.075 (2015).

2 Yan, C. G. *et al.* A comprehensive assessment of regional variation in the impact of head micromovements on functional connectomics. *Neuroimage* **76**, 183-201, doi:10.1016/j.neuroimage.2013.03.004 (2013).

3 Fox, M. D., Zhang, D., Snyder, A. Z. & Raichle, M. E. The global signal and observed anticorrelated resting state brain networks. *Journal of neurophysiology* **101**, 3270-3283 (2009).

4 Hayasaka, S. Functional connectivity networks with and without global signal correction. *Frontiers in human neuroscience* **7**, 880 (2013).

5 Saad, Z. S. *et al.* Trouble at rest: how correlation patterns and group differences become distorted after global signal regression. *Brain connectivity* **2**, 25-32 (2012).

6 Murphy, K., Birn, R. M., Handwerker, D. A., Jones, T. B. & Bandettini, P. A. J. N. The impact of global signal regression on resting state correlations: are anti-correlated networks introduced? **44**, 893-905 (2009).

7 Yang, G. J. *et al.* Altered global brain signal in schizophrenia. **111**, 7438-7443 (2014).
